# Bibliometric analysis of global research on dialectical behavior therapy from 1987 to 2024

**DOI:** 10.3389/fpsyg.2025.1450497

**Published:** 2025-02-20

**Authors:** Kuo Shi, Li-Yi Zhang, Bing-Ling Gao, Ying Qian, Xue-Bing Huang, Jing-Li Yue

**Affiliations:** ^1^Peking University Sixth Hospital, Peking University Institute of Mental Health, NHC Key Laboratory of Mental Health (Peking University), National Clinical Research Center for Mental Disorders (Peking University Sixth Hospital), Beijing, China; ^2^Beijing Jishuitan Hospital, Capital Medical University, Beijing, China

**Keywords:** behavior therapy, Bibliometrics, visualization, DBT, global

## Abstract

**Objective:**

This study explores researches of dialectical behavior therapy (DBT) in mental health to provide an overview of current knowledge landscape and predict future development trends of DBT.

**Method:**

The bibliometric approach was used in the study. Articles on DBT-related research were retrieved from the Web of Science Core Collection (WoSCC) database up to December 31, 2024, and analyzed using VOSviewer and CiteSpace.

**Results:**

A total of 2,723 articles were analyzed. DBT research has grown significantly since the 1990s, with the United States leading in publication volume, citation impact, and academic collaboration. Research is primarily conducted in developed countries like the United States, the United Kingdom, and Germany, with limited contributions from emerging economies. *Cognitive and Behavioral Practice* is the most prolific journal in DBT research. Key topics include borderline personality disorder (BPD), suicide, adolescent interventions, forensic psychiatry, and family therapy. Recently, keywords such as “emotion dysregulation” and “mobile phone” have become research hotspots.

**Conclusion:**

DBT research has evolved from early focus areas like BPD and suicide to studies on emotion dysregulation mechanisms and digital interventions. While the United States dominates the field, expanding participation from emerging countries and strengthening global collaboration could advance DBT research and improve mental health accessibility. This bibliometric analysis provides a global perspective and long-term trend insights, highlighting future directions in neurobiological mechanisms, methodological innovation, and technological integration.

## Introduction

Dialectical behavior therapy (DBT) is one of the leading approaches for treating individuals bearing borderline personality disorder (BPD) and self-harm or suicidal behaviors, which is originally developed by Linehan (Bloom et al., 2012; Lammers et al., 2022; MacPherson et al., 2013). DBT blends cognitive-behavioral approaches with practices embodied by the dialectical thinking of Zen, in which the dialectical balance of acceptance and changes and relationship between normal and abnormal psychology and behavior is emphasized (Liang et al., 2021; Lynch et al., 2006). The standard DBT has multicomponent including weekly individual therapy, weekly group skills training, as-needed between-session telephone coaching and weekly therapist consultation team meetings (Linehan et al., 2015). Individual therapy targets the aforementioned hierarchy of behaviors occurring either in session or reported on the clients record daily card (Rizvi et al., 2013). Groups are devoted to homework review and teaching new skills, i.e., mindfulness, distress tolerance, emotion regulation and interpersonal effectiveness (MacPherson et al., 2013). Telephone coaching calls are encouraged if clients are experiencing suicide or self-injurious urges, they need help utilizing skills or there is a rupture in the therapeutic relationship. Therapist consultation team meetings hold therapists within the therapeutic frame, balance therapists’ interactions with clients, address problems that arise in treatment, increase adherence to DBT principles, and increase therapists’ motivation and capabilities in delivering DBT (MacPherson et al., 2013). These modes can reduce individuals’ dysfunctional behaviors when they are encountering the dysregulated emotion (MacPherson et al., 2013).

Based on the DBT theory, the underlying problem is pervasive emotion regulation (i.e., sensitivity to emotional stimuli, intensity of emotional reactions, and inability to regulate negative affective responses), which leads to impulsive and maladaptive behaviors (Asarnow et al., 2021; DeCou et al., 2019). DBT targets the common underlying dysfunctional emotion regulation among the psychiatric disorders and problem behaviors, such as BPD, depression, anxiety disorders, post-traumatic stress disorder (PTSD), eating disorders (ED), suicidal behaviors, and non-suicidal self-injury (NSSI) (Bohus et al., 2020; Lammers et al., 2022; Liang et al., 2021; Snoek et al., 2020). Studies of DBT for BPD found a low overall dropout rate (27.3%) and moderate before-and-after effect sizes for global outcomes as well as suicidal and self-injurious behaviors (Kliem et al., 2010). DBT is the only treatment with sufficient replication to be considered evidence based for BPD (Binks et al., 2006). DBT prioritizes suicidal behavior and other self-directed violence as the primary treatment targets, indicating its clinical implications on suicidal behavior over suicidal thoughts (DeCou et al., 2019). DBT is effective in reducing binge ED behaviors, however, less efficacy assertions can be made about DBT with those diagnosed with bulimia nervosa or anorexia-nervosa (Bankoff et al., 2012; Ben-Porath et al., 2020). In addition, one of DBT components-skills training group-reduced BPD symptoms and emergency department presentations of people with BPD (Heerebrand et al., 2021). DBT is effective for reducing suicide attempts and NSSI episodes, meanwhile, DBT skills training are more effective than DBT individual therapy, and standard DBT is superior in some areas (Linehan et al., 2015). DBT for adolescent (DBT-A) has been adapted with various psychiatric disorders (i.e., BPD, mood disorders, externalizing disorders, ED, trichotillomania) and problem behaviors (i.e., suicide ideation and behavior, NSSI) across several settings (i.e., outpatient, day program, inpatient, residential, correctional facility) (Kothgassner et al., 2021; MacPherson et al., 2013).

Although multiple reviews and meta-analyses have summarized the application and effectiveness of DBT in clinical settings, as well as its improvements and innovations, mechanism research, adaptation and dissemination, and factors related to skills training and therapist performance (Asarnow et al., 2021; Heerebrand et al., 2021; Linehan et al., 2015; McCauley et al., 2018), as far as we know, studies of DBT have not emerged by means of bibliometrics after many years of advancements to provide an overview of temporal trends in this field. Bibliometrics is a quantitative method to statistically analyze studies to evaluate the impact of institutions, countries and authors, which is widely used to map and describe performance in particular fields, and predict the development of specific fields (Ninkov et al., 2022). To fill this knowledge gap, this study aimed to perform a bibliometric analysis of DBT publications to reveal major contributors, current research status, the research trends and future development prospects from a general perspective.

## Methods

### Data search and retrieval strategy

In this study, the relevant literature was searched and exported in the Web of Science Core Collection (WoSCC) database from inception to December 31, 2024. We selected Thematic Suffix (TS) for retrieval. Specific search strategy was conducted as follows: (1) TS = (dialectical behavior therapy OR Behavior Therapy, Dialectical OR Dialectical Behavior Therapies). (2) Only articles and reviews were included, while other document types such as meeting abstracts, letters, book reviews, and corrections were excluded. (3) All contents, including title, author, abstract, keywords, and cited literature, were recorded. (4) The search was limited to publications in English language and those on the topic.

Two authors independently verified the data collection and entry. Disagreements were resolved by the third author to reach a consensus. After excluding 239 studies that did not meet the inclusion criteria, 631 journals, 2,723 papers, and 77,221cited references from 2,846 institutions across 67 countries were eligible for analysis.

### Data analysis and visualization

VOSviewer (Leiden University, Leiden, The Netherlands, VOSviewer version 1.6.19) and CiteSpace (Drexel University, United States, CiteSpace 6.2.R4) were used to analyze and visualize all the bibliographic data of the selected publications including authors, institutions, journals, countries, references, and keywords. In this study, VOSviewer was used to perform (1) a co-authorship network that explored the authors’, their institutions’ and their countries’ collaboration networks and (2) co-occurrence network that reflected the associations between authors’ keyworks. CiteSpace can obtain quantitative information and discover relevant developments and trends in specific scientific research fields with loaded burst and cluster analyses mode. In this study, it was used to analyze and visualize the keyword co-citation clusters, timeline view of clusters, and the citation bursts. Co-occurrence analysis is a method used to discover patterns and associations of co-occurrence between terms or keywords. By analyzing the frequency and patterns of co-occurrence of terms or keywords in documents, it can reveal the relevance and patterns of association between these terms or keywords. The graphs were created by the abovementioned software.

## Results

### Analysis of publications and citations

**Figure 1:**
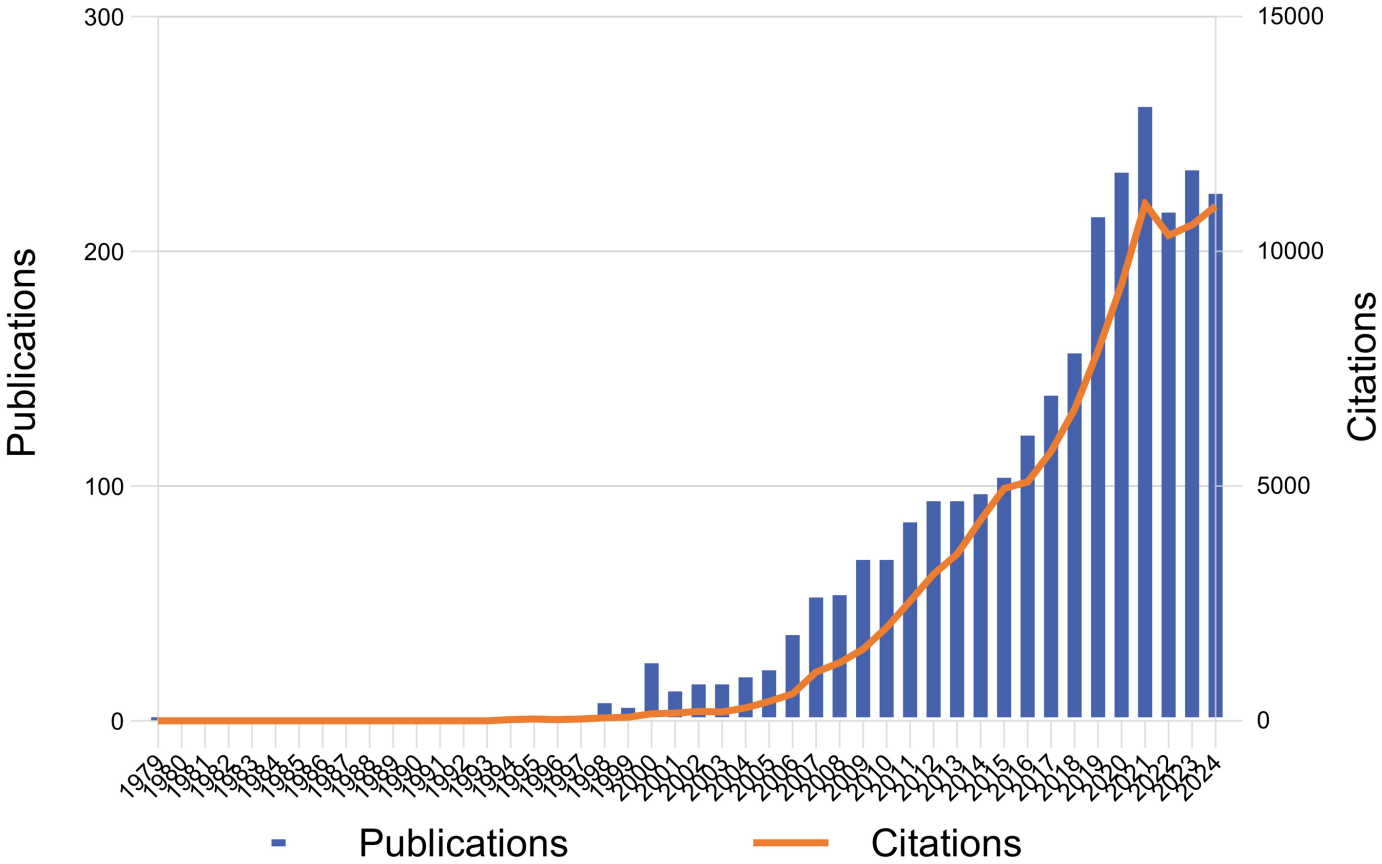
Total number of DBT publications and citations annually from 1987 to 2024.

This study included a total of 2,723 articles identified from the WoSCC database spanning from 1987 to 2024. The number of research articles on DBT has increased rapidly in the past two decades, from 1 publication in 1998 to 249 publications in 2021 (accounting for 11.32% of the total publications). Figure 1 shows the number of annual publications. There was an explosive growth from 2015 (138 publications) to 2021 (249 publications). The other obvious increase was seen from 2007 (53 publications) to 2014 (103 publications). The number of citations showed a rapid growth trend after 2007, reaching the high point in 2021 (11,094 citations). There was a slight drop in the number of citations in 2022 (10,196 citations). From 2022 to 2024, the number of publications and citations tended to level off.

### Analysis of countries

**Figure 2:**
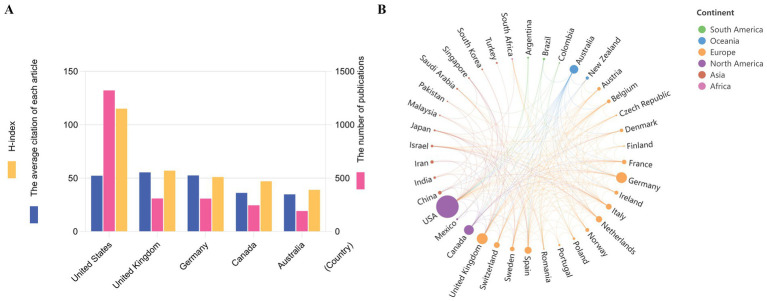
Publications in different countries and co-operations between various countries in DBT research fields. **A** presented countries at the top five international rankings in DBT studies. Countries ranked international top five related to DBT were evaluated according to the number of publications, H index and the average number of citations per article. **B** was the cooperative relationships among countries according to various continents. The size of the points indicates the number of publications, and the weight of the lines indicates the degree of closeness of cooperation.

The total number of publications related to DBT originated from 67 identified countries (the minimum number of studies from a country was over five). Figure 2A shows the number of publications, average citation and H-index in the top five countries. The United States had the highest number with 1,320 articles, accounting for 48.49%. Germany (307, 11.28%), the United Kingdom (276, 10.14%), and Canada (248, 9.11%) ranked second to fourth. The United Kingdom had the highest average citation of each article (68,704 citations). The highest ratio of citation and publication was seen in Sweden (61.88%), the United Kingdom and the United States ranked the second (59.78%) and third (52.05%) respectively. The other top five countries had relatively low ratio of citation and publication, specifically, 47.26% for Netherlands, 40.52% for Germany and 38.41% for Italy (Supplementary Table 1). Figure 2B shows the cooperation network among different countries. United States had close co-operations with Canada, Germany and United Kingdom, and close relationship was seen among United Kingdom and Germany as well. The top five countries with the greatest total link strengths were as follows: United States (370), Germany (208), United Kingdom (200), Canada (143) and Switzerland (120). Countries in Europe, and United States and countries in Europe communicated most, the North America and Europe cooperated more as well, while countries in Africa and South America had no communication with others.

### Analysis of institutions

**Figure 3:**
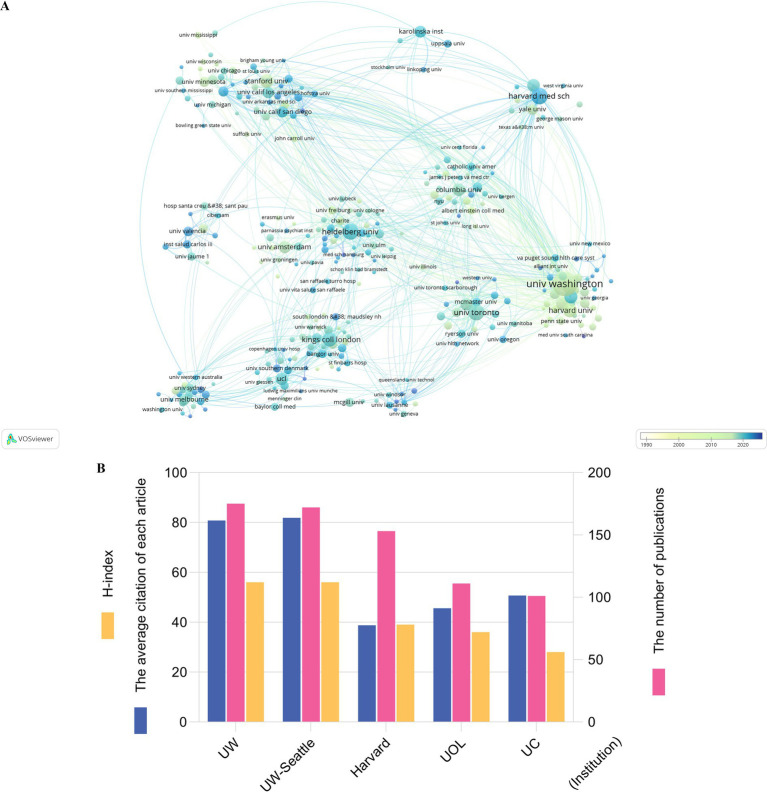
Publications in different institutions and cooperations between various institutions in DBT research fields. **A** showed the time trend of cooperative relationships among institutions. The color represented the annual time period of publications, in which the color from white to blue represented the annual occurrence time from 1987 to 2024. Each point corresponds to an academic institution contributing to DBT research. The size of the point indicates the number of publications by that institution. Larger points represent institutions with more publications. Lines between points signify collaborative relationships between institutions. Thicker lines indicate stronger collaboration or higher co-authorship frequency. The color gradient reflects the temporal distribution of publications. Lighter blue represents earlier collaborations. Darker blue to green represents more recent collaborations. Clusters are formed based on the strength and frequency of collaborations. Institutions within the same cluster often work closely together and share common research themes or focus areas. **B** presented the top five institutions worldwide in the field of DBT research, institutions ranked international top five related to DBT were evaluated according to the number of publications, H index and the average number of citations per article. UW: University of Washington; UW-Seattle: University of Washington Seattle; Harvard: Harvard University; UOL: University of London; UC: University of California System.

The publications in 2,846 identified institutions (the minimum number of publications from an institution was over five) were analyzed by VOSviewer. Figure 3A shows the time trend of cooperative relationships among different institutions. University of Washington, Stanford University, Columbia University, University of Pittsburgh and University of Amsterdam focused on DBT earlier, while King’s College London, University of Melbourne and Heidelberg University began focusing on DBT recently. As Figure 3B shows that University of Washington had the highest number of publications (172), followed by the University of Washington Seattle (169) and Harvard University (154), with 12,826 citations, 12,768 citations and 4,698 citations, respectively. The top five institutions with the greatest total link strengths were as follows: University of Washington (17,391), Duke University (4,949), the University of Toronto (4,457), Heidelberg University (4,083), and Stanford University (3,702). The ratio of citation and publication for University of Washington Seattle was the highest (76.00%), the second for University of Washington (75.45%), which was relatively low for Harvard University (32.63%) (Supplementary Table 2).

### Analysis of researchers

**Figure 4:**
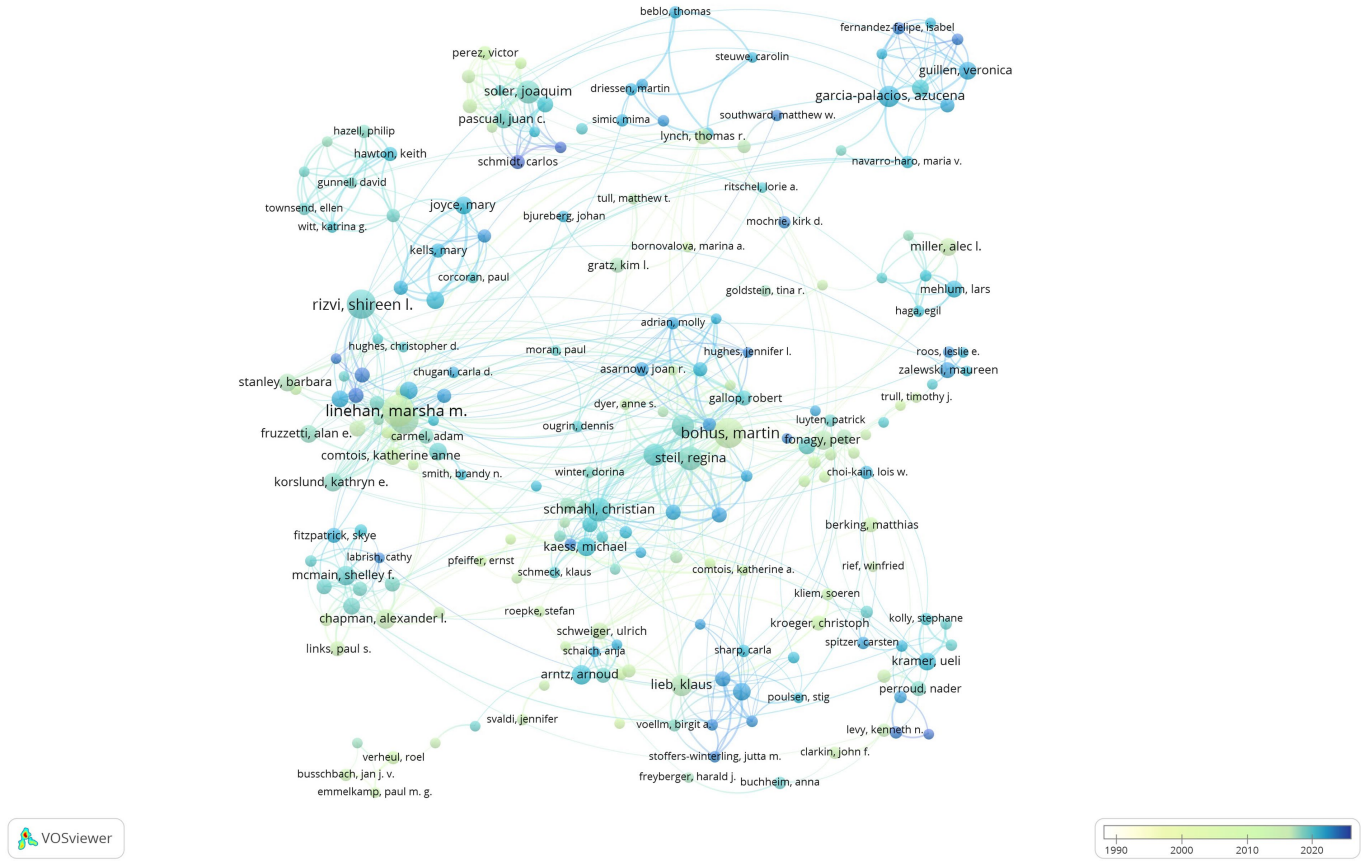
The cooperative relationships of authors who published DBT articles. It showed the time trend of cooperative relationships among authors, and the colors represented the annual time period of the publications, in which the colors from white to blue represented the annual occurrence time from 1987 to 2024. Each point represents an author contributing to DBT research. Larger points indicate authors with more publications or higher influence in the field. Lines signify co-authorship or collaborative relationships between authors. Thicker lines represent stronger collaboration or more frequent co-authorship. Lighter blue indicates earlier collaborations. Darker blue to green indicates more recent collaborations or publication activities. Authors are grouped into clusters based on their co-authorship and collaboration networks. These clusters often reflect research teams or authors with similar research focuses or geographic proximity.

8,915 authors were identified (the minimum number of publications from an author was over five), and their publications were analyzed by VOSviewer. Figure 4 shows the author network of close co-operations, and there were more than 10 cooperative clusters among different researchers. The cooperative network with core author of Schmahl, Christian had a close relationship with the network with core author of Bohus, Martin. The top five authors with the greatest total link strengths are: Bohus, Martin (235 times), Schmahl, Christian (201 times), Linehan, Marsha M. (188 times), Soler, Joaquim (174 times), and Steil, Regina (171 times). Linehan, Marsha M has published the highest number of articles in the DBT field (66 articles), followed by Bohus, Martin (46 articles) and Rizvi, Shireen L. (41 articles) (Table 1). Two partnership clusters with the core authors of Linehan, Marsha M and Bohus, Martin started studies on DBT nearly between 1990 and 2000, and new co-authors are cooperating with authors in these two clusters. New authors network emerged in recent years (about 2020), Marco JH and Garcia-palacios A, Jorgensen MS, Simonsen E and Stoffer-winterling JM, Gillespie C and Flynn D.

**Table 1:** Authors’ information of top 10 publications.

Rank	Authors	Publications	Citations	Citation/publication (%)	H-index	Institution
1	Linehan, Marsha M.	66	10,082	152.76	44	University of Washington
2	Bohus, Martin	46	2,388	51.91	23	Ruprecht Karls University Heidelberg
3	Rizvi, Shireen L.	41	1,270	30.98	15	Rutgers University System
4	Harned, Melanie S.	36	1,747	48.53	19	US Department of Veterans Affairs
5	Steil, Regina	27	1,438	53.26	15	Goethe University Frankfurt
6	Soler, Joaquim	27	982	36.37	14	Hospital of Santa Creu i Sant Pau
7	Pascual, Juan Carlos	25	938	37.52	13	Universitat Autònoma de Barcelona
8	Miller, Alec L.	24	1,440	60	16	Cognit & Behav Consultants
9	Lieb, Klaus	23	2,200	95.65	14	Johannes Gutenberg University of Mainz
10	Comtois, Katherine Anne	23	3,037	132.04	17	University of Washington

### Analysis of journals

The journal was included if the minimum number of citations from a source was over 20 times. There were 631 journals that met the criteria. *Cognitive and Behavioral Practice* published the most articles on DBT with 93 publications (Supplementary Table 3). *Journal of consulting and clinical psychology* had the highest average citations per article (124.46). *Behavior research and therapy* published the most articles on DBT and had the largest number of total citations with 3,818 (Supplementary Table 4).

### Analysis of co-cited references

**Figure 5:**
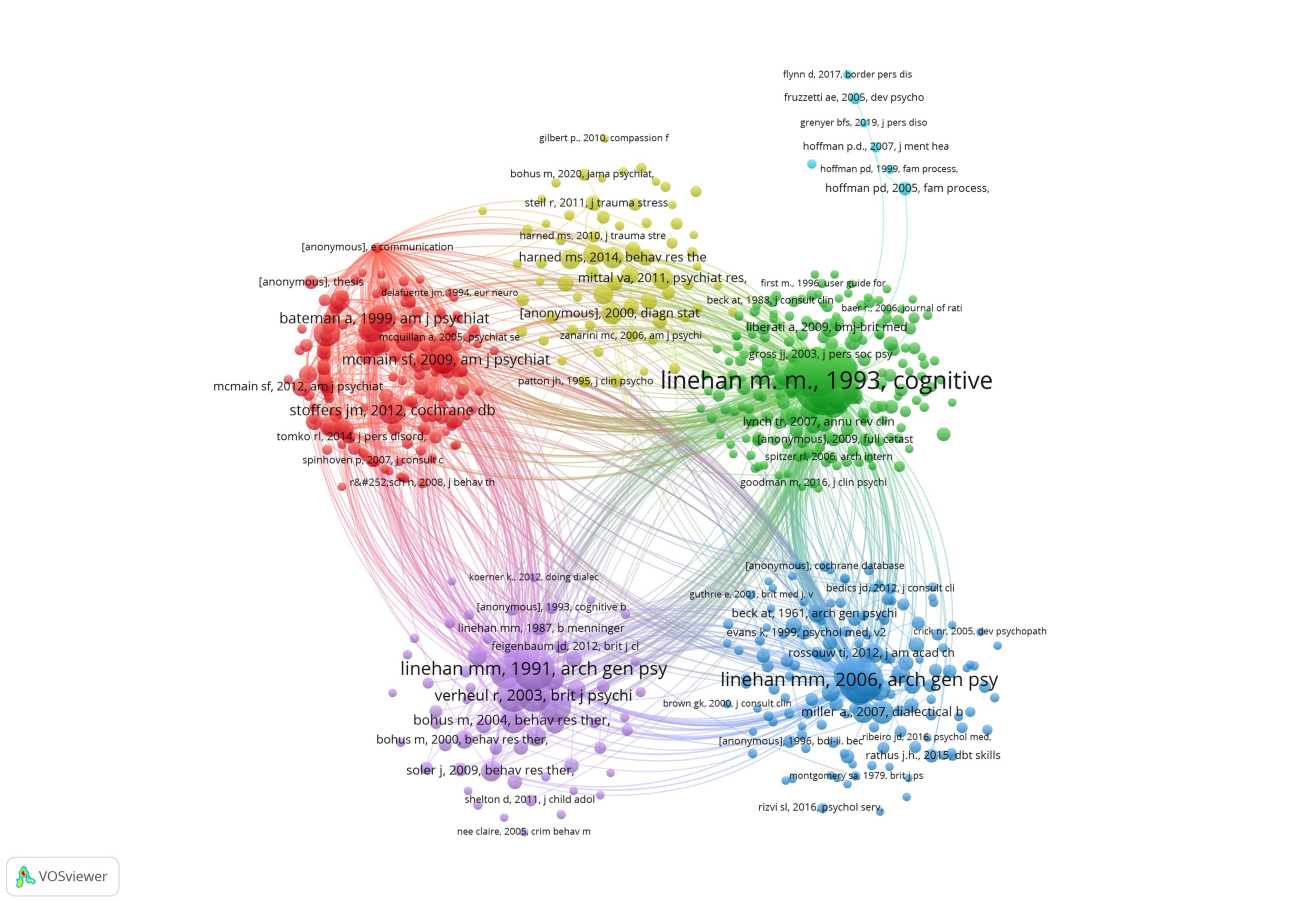
Visualization network of co-cited references regarding DBT articles, and a color represented a cluster. Each point represents a cited article in DBT research. Larger points indicate articles with higher citation frequencies, suggesting their influence and importance in the field. Lines signify co-citation relationships, indicating that two articles are frequently cited together. Thicker lines represent stronger co-citation connections. Points are grouped into clusters based on their co-citation relationships, with each cluster representing a thematic focus or subfield in DBT research. Different colors highlight distinct research themes or areas of emphasis.

A total of 675 references (the minimum number of citations of a reference was over 20 times) were analyzed by using VOSviewer. Figure 5 showed the visualization network of clusters with the colors representing clusters, and there were six clusters of co-cited references. Supplementary Table 5 shows the top 10 of 2,723 tabulated references which the number of co-cites was more than 1,000. Among the top 10 most frequently co-cited articles, four of these ten articles listed were published in journals with impact factors (IF) more than 10. Five of these articles are randomized trials. Two of these articles focused on adolescents’ suicide and self-harm behaviors, while others mostly focused on BPD.

### Analysis of keywords

**Figure 6:**
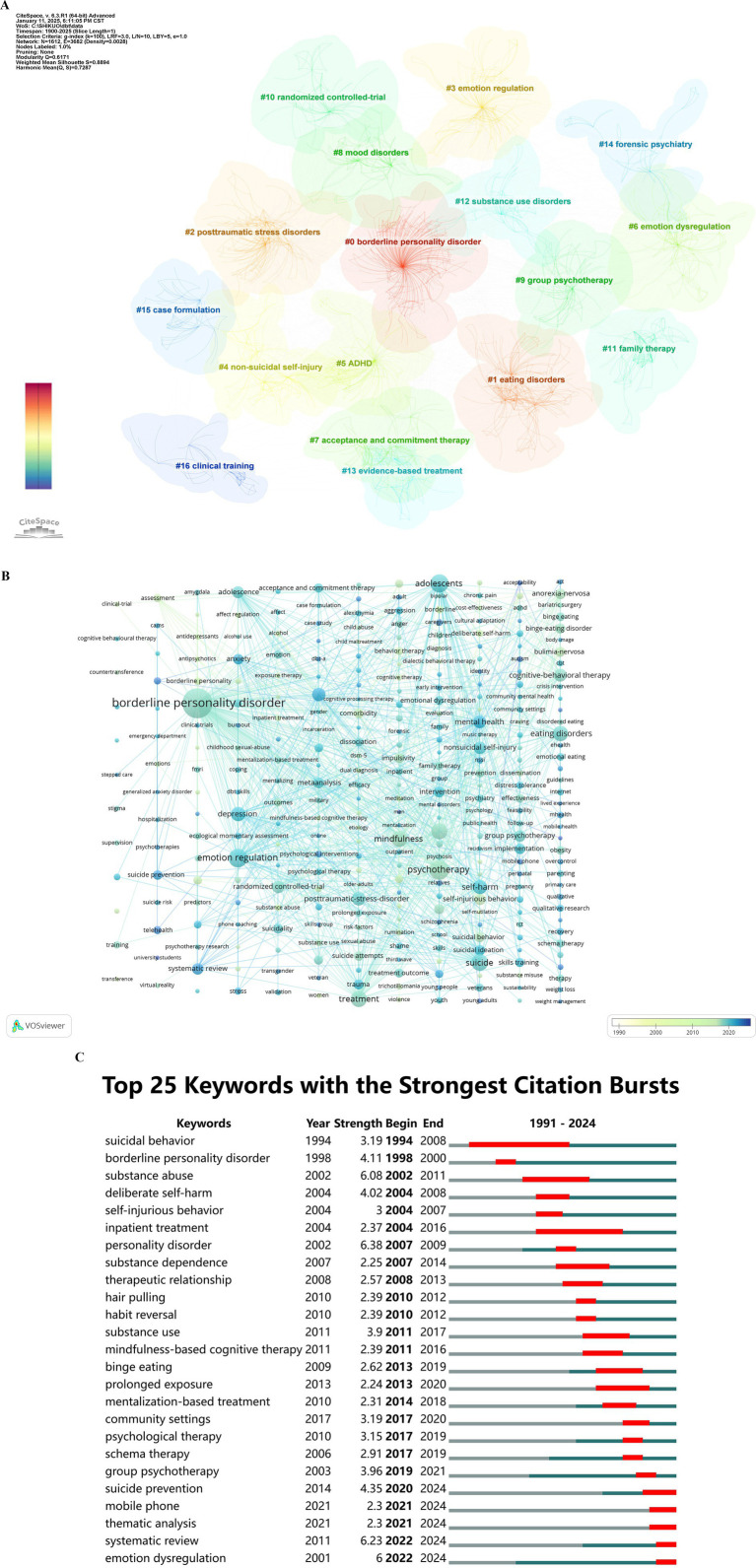
Visualization network of keywords occurring in DBT publications. **A** was cluster diagram of keywords and a color represented a cluster automatically calculated by CiteSpace. Each numbered cluster represents a distinct research focus within the field of DBT. Larger cluster numbers represent smaller or more niche topics. Each point within a cluster corresponds to a specific publication contributing to that topic. The density and size of points indicate the volume and interconnectivity of research within the cluster. Lines connecting points represent co-citation relationships, with denser connections indicating stronger thematic coherence within a cluster. The color scale (red to blue) indicates the temporal distribution of publications within each cluster. **B** was timeline graph of keyword cluster analysis corresponding to **A**. The color represented the annual time period of the publication, in which the color from white to blue represented the annual occurrence time from 1987 to 2024. The size of a point indicates the frequency of the keyword’s occurrence. Larger points represent keywords that appear more frequently in the literature. Lines between points signify co-occurrence relationships between keywords. Thicker and denser lines indicate stronger associations or higher co-occurrence frequencies. Lighter blue indicates keywords appearing in earlier studies (1990s). Darker blue to green indicates keywords associated with more recent research (after 2020). Groups of interconnected nodes form clusters, representing thematic areas or research topics within DBT studies. **C** showed the top 25 with the strongest citation bursts in DBT research. Red bars represent the duration of strong citation bursts for each keyword. Longer red bars indicate sustained academic interest during that period. Blue lines represent the full timeline (1991–2024) of the keyword’s occurrence. Keywords: listing the keywords with significant citation bursts. Year: the first recorded occurrence of the keyword. Strength: the intensity of the citation burst. Higher values indicate stronger academic attention. Begin-End: the start and end years of the citation burst for each keyword.

Analysis of keywords is to determine research areas and hot issues, an important indicator to track scientific development. The keywords (the minimum number of occurrences of a keyword was over five) were analyzed by CiteSpace. A total of 3,602 identified keywords were analyzed, and there were seventeen clusters including BPD, ED, PTSD, Emotion Regulation, NSSI, ADHD, Emotion Dysregulation, Acceptance and Commitment Therapy, Mood Disorders, Group Psychotherapy, Randomized Controlled Trial, Family Therapy, Substance Use Disorders, Evidence-based Treatment, Forensic Psychiatry, Case Formulation, Clinical Training (Figure 6A). Figure 6B further shown the timespan of documents in each cluster. The research on efficacy of medications were studied earliest, mainly in the 1990s. Later, BPD and suicide/self-harm in adolescents were the research hotspots. In recent years, authors mainly focused on mechanisms of DBT and disorders (such as emotion regulation), and intervention strategies. The keywords with the top three occurrences were “suicide,” “emotion regulation” and “psychotherapy,” as shown in Figure 6C. Twenty-five keywords with strong citation bursts in the DBT were identified by CiteSpace and indicated research trends. Citation bursts for keywords appeared as early as 1987. The bursts strength of these 25 keywords ranged from 2.25 to 6.25, and endurance strength was from 2 to 14 years.

## Discussion

To the best of our knowledge, this is the first study using bibliometrics to summarize and describe the current knowledge landscape and predict future development trends of DBT field from 1987 to 2024. Articles on DBT showed a growing trend over the past decades, particularly since 2007 and 2015, respectively. One of the potential reasons for the expansion of the research might be due to the increase on the recognition and occurrence of personality disorders, depression, ED, PTSD, suicide, self-harm behaviors, and high-risk behaviors among adolescents (Bohus et al., 2020; Chen et al., 2021; Kothgassner et al., 2021; Lammers et al., 2022; Leichsenring et al., 2023; Liang et al., 2021; Park and Kim, 2022; Stoffers-Winterling et al., 2022). Another reason might be the recognition that DBT and these psychiatric disorders shared common mechanism of emotion dysfunctions (Asarnow et al., 2021; Ben-Porath et al., 2020). The number of DBT-related publications continued to increase in 2020 and 2021, possibly due to the peak in extensive exploration of this therapy across various diseases and fields. However, the decrease in 2022 and 2023 reflects a transition in DBT research from a phase of rapid growth to a relatively stable stage, as its intervention methods have gradually matured, and its scope of application and research domains have been largely established. This may have led to a gradual reduction in new research topics.

### Regional disparities and cross-cultural adaptation

The regional disparity in DBT research is significant, with a strong concentration in developed countries, particularly the United States. In this bibliometric analysis, most articles were authored by corresponding authors from the United States, United Kingdom, Germany, Canada, and Australia. Similar patterns have been observed in bibliometric studies in other fields, such as schizophrenia and depression (Sun et al., 2022; Wang et al., 2021). This might be influenced by economic status, academic resources, cultural compatibility, and emphasizing on mental health. The United States, supported by its economic capacity and its status as the birthplace of DBT, has become the central hub of this field, forming strong academic collaborations with countries like the United Kingdom, Germany, and Canada. In contrast, developing and underdeveloped regions face constraints due to limited resources and lower societal attention to mental health issues, restricting the scale and impact of DBT research. Additionally, the multidisciplinary nature and high specialization required for DBT further exacerbate the imbalance in research distribution (Kiraz, 2020).

In cross-cultural research on DBT, a systematic review found that half of studies focused on cultural adaptations for people of color and communities in the United States, with most adaptations involving modifications to language, metaphors, methods, and context (Haft et al., 2022). Different countries demonstrated unique localization characteristics during the adaptation process. For example, China adjusted the interpersonal effectiveness module to align with indirect communication styles and collectivist culture (Lammers et al., 2022). India integrated mindfulness skills with traditional yoga and Buddhist practices (Grover et al., 2018). Brazil focused on developing community-based group therapy approaches (Moritz et al., 2021). Enhancing global collaboration, promoting localized adaptations of DBT, and increasing funding and technical support in resource-limited regions are essential to narrow this gap and achieve more balanced global development of DBT research.

### Leading institutions, journal platforms and collaborative opportunities

The University of Washington and Harvard University hold a leading position in this field. This is partly attributed to the fact that DBT originated in the United States (Linehan et al., 2015). Additionally, these institutions maintain close collaborations with European institutions, forming central academic networks. However, emerging countries like China still have limited participation in global DBT research, partly due to language barriers and the dominance of English-language high-impact articles in databases like Web of Science. Analyzing the characteristics of international peer-reviewed journals is helpful for understanding current research trends (Wu et al., 2021; Zhao et al., 2020).

*Cognitive and Behavioral Practice* has become an important platform for DBT research, closely aligning with its focus on innovative applications and clinical practices of cognitive-behavioral therapy. DBT emerged in the early 1990s, and *Cognitive and Behavioral Practice* was founded in 1994, with their timelines nearly coinciding.^1^ The treatment model and practical nature of DBT complement the journal’s orientation, leading to the publication of numerous DBT research articles in this journal. The co-authorship between authors is instrumental in exploring existing collaborations and identifying potential collaborators (Sun et al., 2022). Core authors, such as Linehan, Marsha M and Bohus, Martin, exhibit significant centrality within their clusters, indicating their leadership roles in DBT research. Collaborative networks between core authors, such as Schmahl, Christian, demonstrate close connections, reflecting active academic exchanges and synergies among researchers. In recent years, emerging authors have gradually joined these collaborative networks, showcasing the ongoing vitality and growing appeal of the DBT field.

### Trends and future directions

The co-occurrence analysis of keywords could show the closeness and prevalence of research topics in scientific areas (Deng et al., 2020), while burst detection analysis serves as a vital tool for exploring the evolution of research hotspots. From 1987 to 2024, DBT research focus has undergone significant transformations. Research primarily centered on the treatment effectiveness of DBT in various disorders, such as from 1987 to 2024, DBT research focus has undergone significant transformations. Research primarily centered on the treatment effectiveness of DBT in various disorders, such as BPD, and suicidal and self-injurious behaviors in the early stage (1987–2010) (Binks et al., 2006; Kliem et al., 2010). During the mid-stage (2010–2020), Research also began to emphasize DBT’s application to specific populations, such as tailored interventions for adolescents, Studies expanded to include substance abuse, ED, and therapeutic relationships (Carter et al., 2020; McCauley et al., 2018; Steuwe et al., 2023; Warner and Murphy, 2022). This period highlighted the optimization of DBT techniques in several ways: (1) changes in treatment duration were explored, focusing on optimizing treatment cycles to enhance intervention efficacy (Tomlinson, 2018); (2) adjustments to DBT components were made, with group skills training emerging as an independent therapeutic approach (Hunnicutt Hollenbaugh and Lenz, 2018); (3) improvements to the emotion regulation module were introduced, including enhancements in emotion recognition, labeling, and coping strategies.

In recent stage (2020–2024), research has focused on three major emerging fields, which also represent future directions. (1) Neurobiological Mechanisms: Studies have investigated the neural foundations of emotion dysregulation. Findings indicate that DBT reduces activity in the amygdala, insula, and anterior cingulate cortex while enhancing connectivity between these regions and the prefrontal cortex, medial prefrontal gyrus, and superior temporal gyrus, thereby improving emotional regulation abilities (Schmitt et al., 2016). Additionally, increased gray matter volume in regions such as the subgenual anterior cingulate cortex and superior temporal gyrus underscores DBT’s neurobiological support for emotional regulation and higher-order cognitive functions like mentalization (Mancke et al., 2018). (2) Research Methods: Approaches like thematic analysis and systematic reviews have advanced the systematic summarization and in-depth exploration of DBT research (Beanlands et al., 2021). (3) Technological Applications: The use of mobile devices has significantly enhanced the accessibility and efficiency of interventions by providing instant support, data tracking, and personalized feedback (Rizvi et al., 2011). These evolving hotspots reflect DBT’s multidimensional development in technological integration, methodological diversification, and mechanism exploration, paving the way for future theoretical and practical advancements.

## Strengths and limitations

Compared to traditional randomized controlled trial and systematic reviews, this study offers a broader global perspective and long-term trend insights through multidimensional analyses of key researchers, institutions, journals, and keyword co-occurrence. It also identifies potential directions for future research, including cross-cultural adaptation and technological integration. Additionally, the study emphasizes regional disparities and academic collaboration, underlining the importance of increasing participation from emerging countries and promoting global mental health equity. Utilizing tools like VOSviewer and CiteSpace, the study visually presents research networks and hotspot developments, providing critical support and reference for DBT theoretical innovation and practical optimization. However, the following limitations must be mentioned. Database variation is a limitation of bibliometric analysis. It is widely known that publications from major databases such as Web of Science, PubMed, Embase and the Cochrane Library differ. Therefore, we may omit some publications due to database bias. Although WoSCC is a very authoritative database, some relevant articles, such as articles published in Chinese, might still have been missed when WoSCC was used as the only database. There is a need to pay attention to the latest publications and other non-English publications in daily research. There might be citation bias as well. High citation frequency does not always indicate high research quality. Some studies might be highly cited due to other factors like citation chains, leading to inaccuracies in assessments. Additionally, from the perspective of the methodological rigor of bibliometric analysis, the quality of the article content was not assessed.

## Conclusion

This study utilizes bibliometric analysis to systematically review the evolution of DBT research, highlighting its progression from early core areas such as BPD and suicide/self-injury to studies on emotion dysregulation mechanisms and digital interventions. The United States dominates this field; however, increasing participation from emerging countries and enhancing international academic collaboration could promote the globalization of DBT research and improve accessibility to mental health. This study provides a broader global perspective and long-term trend analysis, forecasting future research directions such as neurobiological mechanisms, methodological diversification, and technological integration.

## Data Availability

The datasets presented in this study can be found in online repositories. The names of the repository/repositories and accession number(s) can be found in the article/Supplementary material.

## Footnotes

^1^ https://www.sciencedirect.com/journal/cognitive-and-behavioral-practice/about/aims-and-scope
