# Supplementary material for: Bibliometric analysis of global research on dialectical behavior therapy from 1987 to 2024
Source: Front Psychol. 2025 Feb 20;16:1450497. doi: 10.3389/fpsyg.2025.1450497 (PMC11882573; doi:10.3389/fpsyg.2025.1450497)
Supplement: Supplementary file 1 [file Table_1.DOCX]

Supplementary

Table 1. Top 10 countries and areas with most publications

| **Rank** | **Country** | **Publications** | **Citations** | **Citations/publication (%)** | **Leading Institute of Each Country (publications)** |
| --- | --- | --- | --- | --- | --- |
| 1 | USA | 1,320 | 68,704 | 52.05 | University of Washington (172) |
| 2 | Germany | 307 | 12,441 | 40.52 | Ruprecht Karls University Heidelberg (81) |
| 3 | England | 276 | 16,500 | 59.78 | University of London (111) |
| 4 | Canada | 248 | 8,858 | 35.72 | University of Toronto (86) |
| 5 | Australia | 192 | 6,639 | 34.58 | University of Melbourne (32) |
| 6 | Spain | 128 | 3,247 | 25.37 | Ciber Centro de Investigacion Biomedica en Red (54) |
| 7 | Netherlands | 104 | 4,915 | 47.26 | University of Amsterdam (45) |
| 8 | Switzerland | 88 | 2,655 | 30.17 | University of Bern (24) |
| 9 | Italy | 86 | 3,303 | 38.41 | Vita Salute San Raffaele University (15) |
| 10 | Sweden | 56 | 3,465 | 61.88 | Karolinska Institutet (38) |

Table 2. Top 10 institutions with most publications

| **Rank** | **Institution** | **Publications** | **Citations** | **Citation/publication (%)** |
| --- | --- | --- | --- | --- |
| 1 | University of Washington | 172 | 12,826 | 75.45 |
| 2 | University of Washington Seattle | 169 | 12,768 | 76 |
| 3 | Harvard University | 154 | 4,698 | 32.63 |
| 4 | University of London | 111 | 4,038 | 42.51 |
| 5 | University of California System | 100 | 4,115 | 47.30 |
| 6 | US Department of Veterans Affairs | 89 | 3,859 | 43.36 |
| 7 | University of Toronto | 86 | 3,026 | 35.19 |
| 8 | Veterans Health Administration | 86 | 3,828 | 44.51 |
| 9 | Harvard Medical School | 84 | 2,279 | 27.13 |
| 10 | Ruprecht Karls University Heidelberg | 81 | 2,522 | 31.14 |

Table 3. Top 10 journals with most publications

| **Rank** | **Journal** | **Article number** | **Citations** | **Citations/article (%)** | **Most cited article** | **Citations of most cited article** |
| --- | --- | --- | --- | --- | --- | --- |
| 1 | Cognitive and behavioral practice | 93 | 2,077 | 22.33 | Naturalistic Evaluation of Dialectical Behavior Therapy-Oriented Treatment for Borderline Personality Disorder | 153 |
| 2 | Journal of clinical psychology | 56 | 2,443 | 43.63 | Self-Injury: A Research Review for the Practitioner | 425 |
| 3 | BMC psychiatry | 53 | 623 | 11.75 | Radically Open-Dialectical Behavior Therapy for Adult Anorexia Nervosa: Feasibility and Outcomes from an Inpatient Program | 94 |
| 4 | Journal of personality disorders | 51 | 1,695 | 33.24 | Dialectical Behavior Therapy: Current Status, Recent Developments, and Future Directions | 138 |
| 5 | Borderline personality disorder and emotion dysregulation | 49 | 550 | 11.22 | Complex PTSD and Borderline Personality Disorder | 83 |
| 6 | Behavior research and therapy | 47 | 3,818 | 81.23 | Efficacy of the Third Wave of Behavioral Therapies: A Systematic Review and Meta-Analysis | 455 |
| 7 | Clinical psychology psychotherapy | 38 | 731 | 19.24 | Fostering Self-Compassion and Loving-Kindness in Patients with Borderline Personality Disorder: A Randomized Pilot Study | 77 |
| 8 | Frontiers in psychology | 34 | 412 | 12.12 | The Zero Suicide Model: Applying Evidence-Based Suicide Prevention Practices to Clinical Care | 139 |
| 9 | Personality disorders theory research and treatment | 34 | 430 | 12.65 | Longitudinal Twin Study of Borderline Personality Disorder Traits and Substance Use in Adolescence: Developmental Change, Reciprocal Effects, and Genetic and Environmental Influences | 52 |
| 10 | Journal of consulting and clinical psychology | 26 | 2,197 | 124.46 | Change in Attachment Patterns and Reflective Function in a Randomized Control Trial of Transference-Focused Psychotherapy for Borderline Personality Disorder | 558 |

Table 4. Top 10 co-citation journals

| **Rank** | **Journal** | **Citations** | **Total Link Strength** | **IF** | **JCR** |
| --- | --- | --- | --- | --- | --- |
| 1 | American journal of psychiatry | 5,403 | 427,300 | 15.1 | Q1 |
| 2 | Journal of consulting and clinical psychology | 3,593 | 277,733 | 4.5 | Q1 |
| 3 | Archives of general psychiatry | 3,504 | 264,320 | 15 | Q1 |
| 4 | Behavior research and therapy | 3,426 | 257,479 | 4.2 | Q1 |
| 5 | Journal of personality disorders | 3,308 | 266,845 | 2.1 | Q3 |
| 6 | Journal of the American academy of child and adolescent psychiatry | 2,380 | 197,494 | 9.2 | Q1 |
| 7 | British journal of psychiatry | 2,310 | 216,887 | 8.7 | Q1 |
| 8 | International journal of eating disorders | 2,195 | 161,417 | 4.7 | Q1 |
| 9 | Journal of clinical psychology | 1,759 | 130,859 | 2.5 | Q2 |
| 10 | Behavior therapy | 1,752 | 127,113 | 3.4 | Q1 |

Table 5. Top 10 co-cited references

| **Rank** | **Counts** | **Year** | **Author** | **Title** | **Journal** | **IF** | **JCR** |
| --- | --- | --- | --- | --- | --- | --- | --- |
| 1 | 129 | 2019 | DeCou CR | Dialectical Behavior Therapy Is Effective for the Treatment of Suicidal Behavior: A Meta-Analysis | Behavior therapy | 3.4 | Q1 |
| 2 | 127 | 2006 | Linehan MM | Two-Year Randomized Controlled Trial and Follow-Up of Dialectical Behavior Therapy vs Therapy by Experts for Suicidal Behaviors and Borderline Personality Disorder | Archives of general psychiatry | 15 | Q1 |
| 3 | 121 | 2015 | Linehan MM | Dialectical Behavior Therapy for High Suicide Risk in Individuals with Borderline Personality Disorder: A Randomized Clinical Trial and Component Analysis | JAMA psychiatry | 22.5 | Q1 |
| 4 | 120 | 2017 | Cristea IA | Efficacy of Psychotherapies for Borderline Personality Disorder: A Systematic Review and Meta-Analysis | JAMA psychiatry | 22.5 | Q2 |
| 5 | 105 | 2018 | McCauley E | Efficacy of Dialectical Behavior Therapy for Adolescents at High Risk for Suicide: A Randomized Clinical Trial | JAMA psychiatry | 13.3 | Q1 |
| 6 | 105 | 2020 | Storebo OJ | Psychological Therapies for People with Borderline Personality Disorder | Cochrane database of systematic reviews | 8.8 | Q1 |
| 7 | 99 | 2011 | Mittal VA | Diagnostic and Statistical Manual of Mental Disorders | Psychiatry research | 4.2 | Q1 |
| 8 | 75 | 2012 | Stoffers JM | Psychological Therapies for People with Borderline Personality Disorder | Cochrane database of systematic reviews | 8.8 | Q1 |
| 9 | 69 | 2014 | Mehlum L | Dialectical Behavior Therapy for Adolescents with Repeated Suicidal and Self-Harming Behavior: A Randomized Trial | Journal of the American academy of child & adolescent psychiatry | 9.2 | Q1 |
| 10 | 65 | 2003 | Verheul R | Dialectical Behavior Therapy for Women with Borderline Personality Disorder: 12-Month, Random Clinical Trial in the Netherlands | The british journal of psychiatry | 8.7 | Q1 |
